# Short-Term Zinc Supplementation Stimulates Visceral Adipose Catabolism and Inflammation in Mice

**DOI:** 10.3390/nu16213719

**Published:** 2024-10-30

**Authors:** Xiaohua Huang, Dandan Jiang, Yingguo Zhu, Zhengfeng Fang, Bin Feng

**Affiliations:** 1 Meat Processing Key Laboratory of Sichuan Province, College of Food and Biological Engineering, Chengdu University, Chengdu 610106, China; hxh3028@163.com; 2 Animal Nutrition Institute, Sichuan Agricultural University, Chengdu 611130, China; dandanjiang17@163.com (D.J.); yingguo_zhu@163.com (Y.Z.); zfang@sicau.edu.cn (Z.F.); 3 Key Laboratory of Animal Disease-Resistant Nutrition of Ministry of Education, Sichuan Agricultural University, Chengdu 611130, China; 4 Key Laboratory for Food Science and Human Health, College of Food Science, Sichuan Agricultural University, Ya’an 625014, China

**Keywords:** zinc, adipose tissue, lipolysis, insulin, F4/80, inflammation

## Abstract

Background: Zinc (Zn), a fundamental trace element in human biology, exhibits pivotal roles in sustaining vital physiological processes and regulating metabolic homeostasis. Insufficient zinc intake has been linked to deleterious consequences on growth, reproductive functions, metabolic activities, and immune responses in both humans and animals. Oral zinc supplementation is usually performed to meet zinc requirement. Previous studies have shown that long-term supplementation of zinc in mice impaired AKT signaling and induced adipocyte hypertrophy in visceral adipose tissue. Methods: The presented study was conducted to investigate the role and mechanism of short-term zinc supplementation on lipids metabolism. Zinc sulfate was supplemented in the drinking water of C57/BL6J male mice at 30 ppm or 90 ppm for one week. Water consumption, food intake, and body weight were analyzed, adipose tissue and serum profile of metabolites were investigated, and the key genes related to lipid metabolism were analyzed. Results: Short-term zinc supplementation decreased visceral adipose tissue weight and adipocyte size compared to the control group, but no difference was observed in food intake, water consumption, and body weight between the two groups. Further studies revealed that short-term zinc supplementation significantly increased the serum insulin level while decreasing the serum NEFA content. In addition, zinc supplementation increased the expression of *Atgl* and *Hsl* in the visceral adipose tissue compared with the control mice. Furthermore, the phosphorylation level of HSL and protein level of PPARg in the epididymal adipose tissue increased by zinc supplementation compared with the control mice. In comparison, the protein level of FASN was down-regulated by short-term zinc supplementation in the epididymal adipose tissue, although the expression of lipogenic genes was not changed. The expression of *F4/80* and *Tnfa* were increased in zinc-supplemented adipose tissue as compared with the control group. Conclusions: Our findings suggest that short-term zinc supplementation might reduce fat deposition by enhancing lipolysis in mice. Future studies could focus on the effect of intermittent zinc supplementation on fat reduction in both animal models and humans.

## 1. Introduction

Micronutrient deficiency may result in impaired health, including retarded growth, increased risk of acute infectious disease, birth defects, and even death [1,2]. Recently, epidemiological research has shown that people in a “vulnerable period” have a higher demand for micronutrients, especially vegetarians, infants, young children, adolescents, elderly individuals, and pregnant and lactating women [3,4]. Increasing the micronutrient intake of populations by diet supplementation or functional food has been shown to reduce the burden on the mother and morbidity and mortality of the child [5,6]. 

Zinc is recognized as a vital trace element that influences various physiological processes of humans and animals, including development, immunity, cognitive, reproductive, oxidative stress, and metabolism [7,8,9]. Remarkably, zinc deficiency aggravates obesity-related metabolic diseases, including hyperlipidemia, insulin resistance, inflammation, and hyperglycemia [10,11]. Zinc supplementation could improve blood glucose levels in individuals with diabetes, and it is more significant in elderly individuals [8,12]. On the other hand, zinc deficiency increases the concentrations of glucose, cholesterol, and triglycerides in streptozotocin (STZ)-induced diabetic rats [13], while increasing leptin production and stimulating macrophage recruitment in adipose tissue in obese mice [14]. Individuals fulfill their nutritional requirements by incorporating zinc-rich foods into their diet, including various types of meat (pork, chicken, lamb, etc.), vegetables (broccoli, carrots, spinach, etc.), and seafood options (kelp, salmon, oysters, etc.), as well as fruits and nuts [15]. However, despite the global accessibility of these foods, zinc deficiency remains prevalent, particularly in developing nations, indicating that malnutrition or other biological and physiological factors, not solely attributed to the country’s economic status, contribute significantly to this widespread phenomenon [1]. Therefore, maintaining adequate zinc intake is crucial for optimal health. In addition, the bioavailability and functional outcomes of zinc are subject to the influence of the food matrix or the specific zinc formulation utilized in supplements. When administered in supplemental forms, zinc must traverse the intestinal barrier and be absorbed into the systemic circulation for effective utilization. Zinc sulfate (ZnSO_4_) has traditionally been employed as a reference compound for zinc supplementation owing to its favorable bioavailability and cost-effectiveness.

Adipose tissue is the primary storage depot for lipids in the human body. Excessive accumulation of lipids in adipose tissue constitutes the morphological foundation of obesity. Meanwhile, the adipose tissue is also the main organ involved in energy metabolism. Imbalances in glucose and lipid metabolism within the body are the leading causes of metabolic disorders [16,17]. When energy intake is in excess, the adipose tissue synthesizes and stores triglycerides (TAG) using glucose, which is known as lipogenesis. Several key enzymes, including fatty acids synthetase (FASN), stearoyl CoA desaturase 1 (SCD1), and acetyl-CoA carboxylase (ACC1), are involved in the process of lipogenesis. The expression of these key enzymes is upregulated by sterol regulatory element binding transcription factor 1 (SREBF1) and peroxisome proliferator proliferator-activated receptor gamma (PPARg) [18]. On the contrary, TAG can be broken down, known as lipolysis, which is a crucial process in the body that involves the breakdown of stored fats into smaller components for energy production. During lipolysis, adipose triglyceride lipase (ATGL) and hormone-sensitive lipase (HSL) are activated. These enzymes work together to hydrolyze triglycerides into glycerol and fatty acids. Fatty acids can be then broken down into acetyl-CoA, which is finally used to synthesize ATP, the body’s primary energy source [19].

Our previous study found that chronic zinc supplementation impaired AKT signaling and induced adipocyte hypertrophy in perirenal adipose tissue [20]. In the current study, we hypothesized that short-term zinc supplementation might benefit lipids metabolism status in adipose tissue. Mice were supplemented with ZnSO_4_ in the drinking water for one week. The results showed that short-term zinc supplementation decreased visceral adipose tissue weight and adipocyte size compared to the control group and stimulated the expression of lipolytic genes and proteins in the visceral adipose tissue.

## 2. Materials and Methods

### 2.1. Animal Study

Animal protocols were reviewed and approved by the Animal Care and Use Committee of Sichuan Agricultural University (SICAU-2021-078, 8 March 2021). All animal studies were carried out under the guide of Use and Care of Laboratory Animals. Five-week-old C57BL/6J mice were obtained from GenPharmatech (Chengdu, China). The mice were housed in a pathogen-free environment with a constant temperature of 22 °C and humidity of 60%. A total of 9 cages of male mice (2 mice per cage) were randomly split into 3 groups based on comparable average body weight at the age of 15 weeks with 3 cages (6 mice) per group. The control group received spring water (zinc content was less than 0.01 mg/L), while the other two groups were given 30 ppm or 90 ppm zinc-supplemented spring water (132.4 mg/L or 397.2 mg/L zinc sulfate heptahydrate (Z0251, Sigma, Shanghai, China)) for one week. All mice were provided a regular chow diet according to AIN93 (Dossy Experimental Animals Co., Ltd., Chengdu, China. Zinc content was 38.3 mg/kg) [20], and had free access to water. Water consumption and food intake were recorded. One week later, mice were weighed and euthanized with CO_2_ under fed status. Blood was collected by cardiac exsanguination, and serum was stored at −20 °C for further analysis. Liver, perirenal, epididymal, and subcutaneous adipose tissues were removed from the body with delicate scissors. All tissue from the liver, perirenal, epididymal, and subcutaneous adipose tissues were collected, and non-fat tissue was removed from the adipose tissues. The liver was dried with absorbent tissue paper to remove residual blood. Fresh tissues were then weighed, flash-frozen in liquid nitrogen, and stored at −80 °C for further analysis.

### 2.2. Analysis of Serum Zinc Content

Zinc concentration in the serum was measured with a zinc detection kit (E011, Nanjing Jiancheng Bioengineering Institute, Nanjing, China) according to the manufacturer’s instructions.

### 2.3. Analysis of the Metabolite Profiles and Hormone Concentration in Serum

The serum levels of glucose, high-density lipoprotein cholesterol (HDL-C), low-density lipoprotein cholesterol (LDL-C), triglycerides (TAG), non-esterified fatty acids (NEFA), and total cholesterol (TC) were measured on an automatic biochemical analyzer (7020, HITACHI, Tokyo, Japan) with the respective assay kits from Kehua Bio-Engineering (Shanghai, China). The serum insulin levels were measured using a mouse insulin ultrasensitive ELISA kit (80-INSMSU, ALPCO, Salem, MA, USA) according to the manufacturer’s instructions.

### 2.4. Histology Staining

For H and E staining, adipose tissue samples were fixed, embedded in paraffin, and subsequently sectioned into 4 μm slices using a precision microtome (RM2016, Leica, Shanghai, China). The sections were rigorously dehydrated through graded alcohols, immersed in hematoxylin for 5 min, rinsed thoroughly with double distilled water (ddH_2_O), and then counterstained with eosin for 2 min, followed by dehydrating again and mounting onto slides. Imaging software (NIS-Elements F3.2, v4.60, Nikon, Tokyo, Japan) was used to capture the images on a microscope (TS100, Nikon, Tokyo, Japan) with a CCD (DS-U3, Nikon, Tokyo, Japan).

Cell area measurements were conducted using the ImageJ software (v1.49, National Institutes of Health, Bethesda, MD, USA). In brief, 16 images obtained from four mice per group were analyzed, and within each image, 10 representative cells of the average cell size were selected for calculating the mean cell size. The cumulative cell area of 160 cells, spanning four mice in each tissue group, was then utilized to determine the average cell area for that specific group.

### 2.5. RNA Extraction and Real-Time PCR (qRT-PCR)

RNA extraction and real-time PCR procedures were conducted following previously documented protocols [21]. In summary, adipose tissues were grinded in liquid nitrogen, and 100 mg tissue was used for total RNA extraction using TRIzol reagent (Invitrogen, Shanghai, China). The quality of the extracted RNA was evaluated via agarose gel electrophoresis, and its concentration was measured using a spectrophotometer (NanoDrop 2000, Thermo Fisher Scientific, Shanghai, China). Subsequently, complementary DNA (cDNA) synthesis was achieved using a reverse-transcription PCR kit (RR047A, Takara, Dalian, China). Real-time PCR analysis was conducted with Power SYBR Green RT-PCR reagents (4367659, Thermo Fisher Scientific, Shanghai, China) on a quantitative-PCR machine (7900HT, ABI, Carlsbad, CA, USA). For each PCR reaction, the following reagent concentrations were optimized: 300 nM forward primer, 300 nM reverse primer, and 20 ng cDNA sample. The PCR conditions were as follows: 95 °C for 10 min for 1 cycle, followed by 40 cycles of 95 °C for 15 s and 60 °C for 1 min. B-actin (*Actb*) was used as the reference gene, and the 2^−ΔΔCt^ method was employed to quantify the levels of gene expression. The primer sequences utilized in this study are presented in Table 1.

### 2.6. Western Blot Analysis

The protein extraction procedure was conducted according to previously documented protocols [21]. Briefly, adipose tissue powder was homogenized using a homogenizer in a protease inhibitor cocktail (4693116001, Roche, Mannheim, Germany) supplemented cell lysis buffer (Beyotime Biotechnology, Shanghai, China). Subsequently, 30 μg of total protein was separated on a polyacrylamide gel, followed by electro-transferring onto PVDF membranes. The PPARg (2443), pAKT S473 (4060), AKT (4691), and pHSL (3891) antibodies were purchased from Cell Signaling Technology (Shanghai, China); GAPDH (abs132004) antibody was obtained from Absin Biotechnology Company (Shanghai, China). After thorough washing, the membranes were incubated with appropriate horseradish peroxidase-conjugated secondary antibodies (7074 and 7076, Cell Signaling Technology) for 1 h. After additional washing, membranes were incubated with an ECL western blotting detection reagent (1705060, Bio-Rad, Hercules, CA, USA). Then, the protein signals were detected on a Molecular Imager ChemiDoc XRS+ System (Bio-Rad).

### 2.7. Statistical Analysis

The data were analyzed with SAS 9.3 software (SAS Institute Inc., Cary, NC, USA). Firstly, the univariate test was performed to examine the normality and homogeneity of data variances. For normally distributed data, an independent *t*-test was employed to compare differences between the two groups. One-way analysis of variance (ANOVA) procedure followed by Tukey’s multiple range was applied to analyze the difference between the three groups. The results are presented as mean ± SE. *p*-Values less than 0.05 were considered statistically significant.

## 3. Results

### 3.1. Short-Term Zinc Supplementation Decreased Fat Deposition in Mice

**Figure 1:**
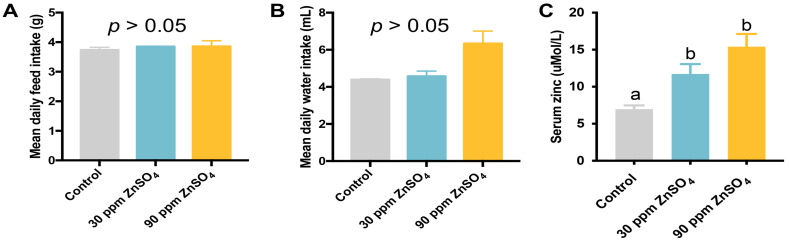
Food intake and water consumption of mice. A total of 18 mice were housed in 9 cages with 2 mice per cage. The initial food weight and water volume of every cage were recorded at the beginning of the study when the mice were 15 weeks of age. Seven days later, the left food weight and water volume were measured. Food intake and water consumption were calculated as follows: (initial food weight − left food weight)/7/2 or (initial water volume − left water volume)/7/2. (**A**) Weight of food intake (N = 3 per group). (**B**) Volume of water consumption (N = 3 per group). (**C**) Serum concentration of zinc (N = 6 per group). Data are shown as mean ± SE. ^a,b^ Columns with different superscript letters mean significant differences (*p* < 0.05).

To explore the effect of short-term zinc supplementation on lipids metabolism, 15-week-old C57/BL6J male mice were given spring water, 30 ppm, or 90 ppm zinc supplemented spring water for one week. The results showed that during the treatment, the food intake (Figure 1A) and water consumption (Figure 1B) were not changed by short-term zinc supplementation compared with the control mice. However, zinc supplementation significantly increased serum concentration of zinc compared to the control (Figure 1C).

Though the body weight and liver weight were not changed, the tissue weight and weight index of the perirenal and subcutaneous fats were decreased by short-term zinc supplementation compared to the control mice, while only the weight index of the epididymal fat decreased (Table 2).

**Figure 2:**
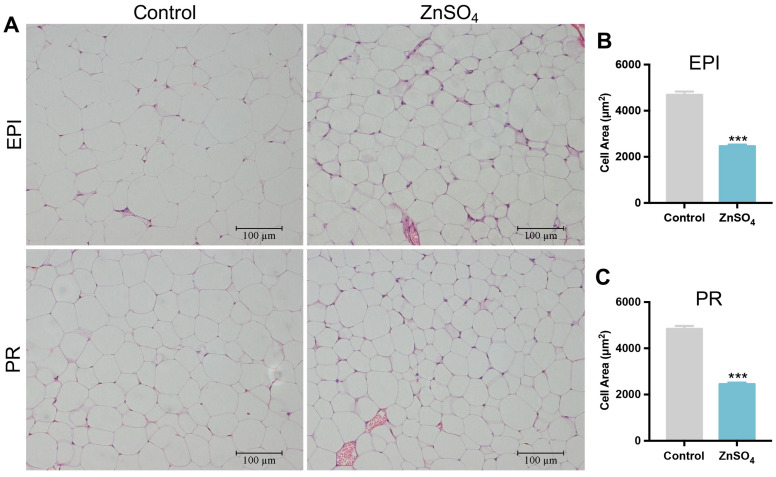
Histological analysis of adipose tissue. (**A**) H and E staining images for epididymal and perirenal adipose tissues; (**B**) Adipocyte cell area of epididymal adipose tissue (N = 160 cells from four mice for each group); (**C**) Adipocyte cell area of perirenal adipose tissue (N = 160 cells from four mice for each group). Bars equal to 100 μm. Data are shown as mean ± SE. ZnSO_4_, 30 ppm zinc supplemented group. *** *p* < 0.001 ZnSO_4_ vs. Control.

Because 90 ppm zinc supplementation had a similar effect on the serum concentration of zinc and weight index of the white fat with 30 ppm zinc supplementation, the following indicators were analyzed only with 30 ppm zinc-supplemented group and control group. Hematoxylin and eosin (H and E) stain showed that the cell area of adipocytes in 30 ppm zinc-supplemented mice decreased by 50.6% and 52.4% in the perirenal and epididymal adipose tissues, respectively, compared with those in the control mice (Figure 2A–C). These data suggest that short-term zinc supplementation could decrease visceral adipose tissue weight and adipocyte size in mice.

### 3.2. Short-Term Zinc Supplementation Induced Hyperglycemia and Hyperinsulinism in Mice

**Figure 3:**
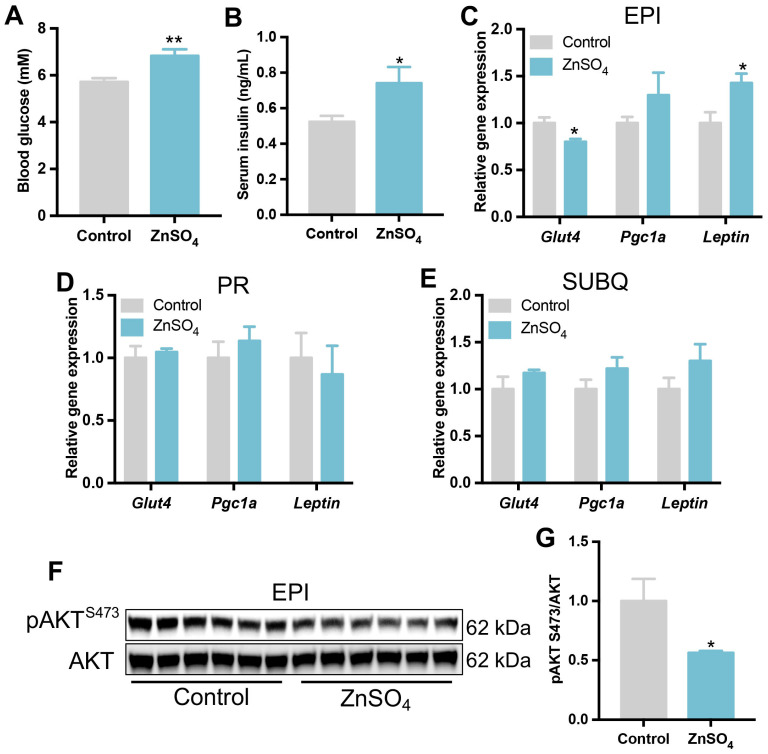
Short-term zinc supplementation induced hyperglycemia in mice. (**A**) Blood glucose level at harvest. (**B**) Insulin concentration in serum. Gene expression level of *Glut4*, *Pgc1a*, and *Leptin* in the epididymal (**C**), perirenal (**D**), and subcutaneous (**E**) adipose tissues. (**F**,**G**) Phosphorylation levels of AKT at S473 in the epididymal adipose tissue. (N = 6 for each group). Data are expressed as Mean ± SE. ZnSO_4_, 30 ppm zinc supplemented group. * *p* < 0.05, ** *p* < 0.01.

Blood glucose levels were then evaluated in the mice. The results revealed that zinc-supplemented mice showed higher blood glucose levels than the control mice under the fed state (Figure 3A). Moreover, serum insulin concentration significantly increased by short-term zinc supplementation compared with the control mice (Figure 3B). Further studies indicated that the gene expression of glucose transporter 4 (*Glut4*), the main regulator for glucose uptake in adipocyte, decreased in the epididymal adipose tissue of zinc-supplemented mice compared with the control group (Figure 3C). The expression of adipokine gene *Leptin* was increased in the epididymal adipose tissue of zinc-supplemented mice compared with that in the control mice (Figure 3C). However, the mRNA levels of *Glut4*, *Pgc1a*, and *Leptin* were not changed by zinc supplementation in perirenal or subcutaneous adipose tissues (Figure 3D,E). Western blot analysis showed that short-term zinc supplementation impaired the phosphorylation level of AKT at Ser473 in the epididymal adipose tissue compared with the control group (Figure 3F,G). These data suggest that short-term zinc supplementation might impair the insulin sensitivity of adipose tissue and thus induce hyperglycemia in mice.

### 3.3. Short-Term Zinc Supplementation Reduced Serum NEFA Concentration

**Figure 4:**
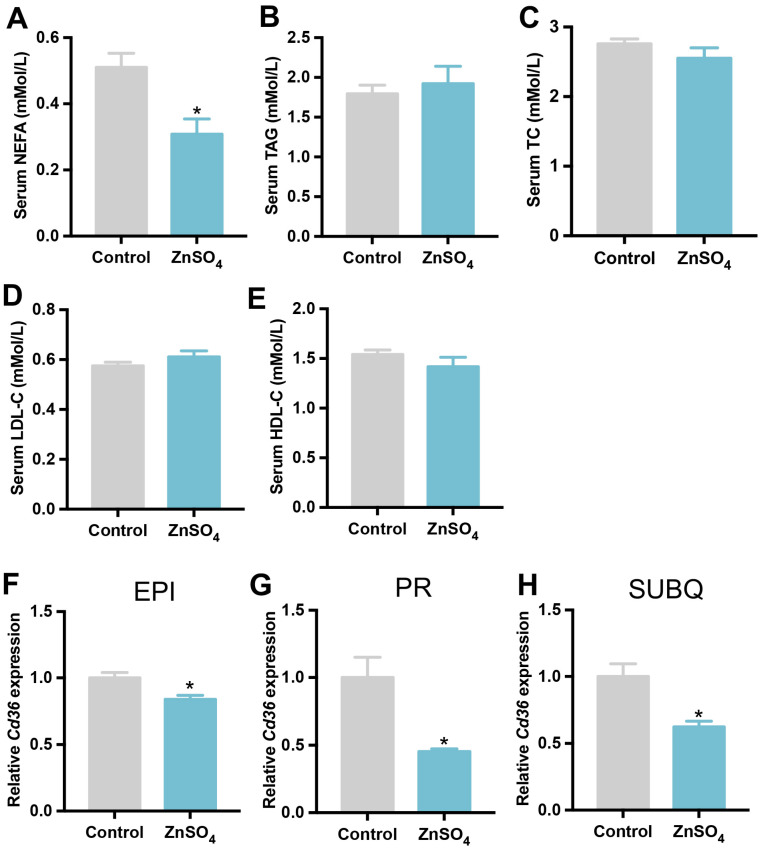
Lipid profiles in the serum. C57/BL6J male mice were supplemented with 30 ppm ZnSO_4_ or control for one week. Serum and adipose tissues were harvested at fed state 7 days post zinc supplementation. (**A**) NEFA concentration in the serum. (**B**) TAG concentration in the serum. (**C**) TC concentration in the serum. (**D**) LDL-C concentration in the serum. (**E**) HDL-C concentration in the serum. The expression level of *Cd36* in the epididymal (**F**), perirenal (**G**), and subcutaneous (**H**) adipose tissues (N = 6 for each group). Data are expressed as mean ± SE. ZnSO_4_, 30 ppm zinc supplemented group. * *p* < 0.05.

Serum lipid profiles were then investigated, which indicated that serum NEFA concentration was lower in the zinc-supplemented mice than that of the control mice (Figure 4A). However, serum levels of TAG, TC, LDL-C, and HDL-C were similar between the zinc-supplemented mice and control mice (Figure 4B–E). Of the fatty acid transporter genes, the mRNA level of fatty acid translocase (*Cd36*) was down-regulated in the epididymal, perirenal, and subcutaneous adipose tissues (Figure 4F–H), by zinc supplementation compared with control. These data suggest that short-term zinc supplementation might reduce circulating NEFA concentration by impairing fatty acid exportation in adipose tissue.

### 3.4. Short-Term Zinc Supplementation Impaired Lipogenesis in the Epididymal Adipose Tissue

**Figure 5:**
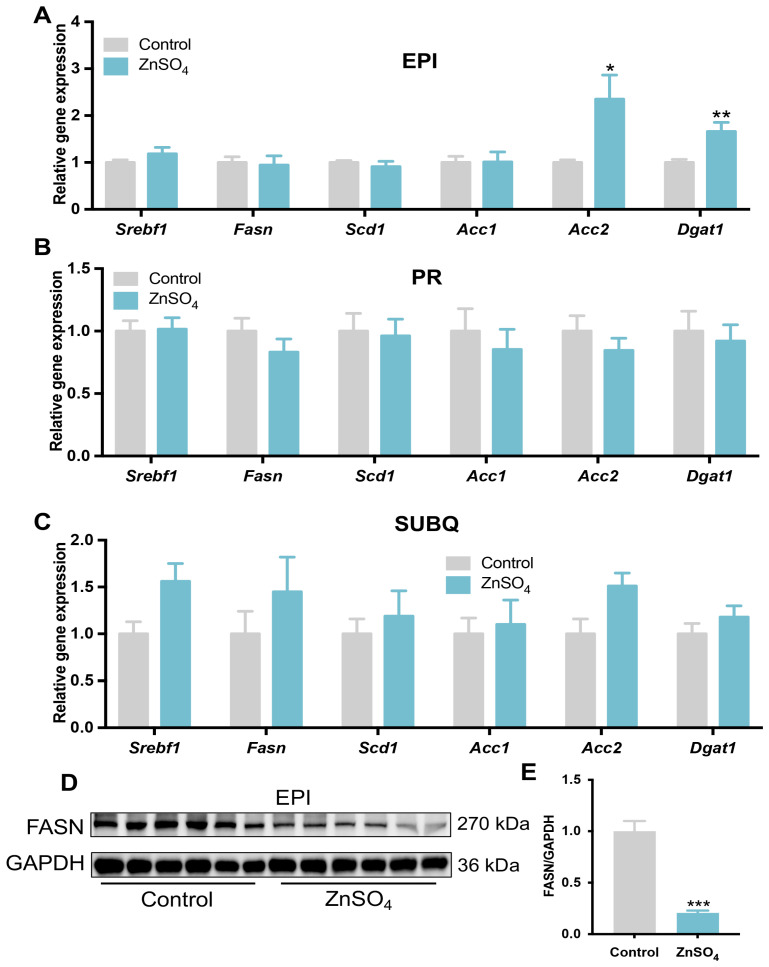
Lipid anabolism genes and protein expression in adipose tissues. The expression level of lipogenesis genes in the epididymal (**A**), perirenal (**B**), and subcutaneous (**C**) adipose tissues. (**D**,**E**) The protein levels of FASN in the epididymal adipose tissue (N = 6 for each group). Data are expressed as Mean ± SE. ZnSO_4_, 30 ppm zinc supplemented group. * *p* < 0.05, ** *p* < 0.01, *** *p* < 0.001.

The accumulation of triglycerides in the adipocytes can be affected by lipogenesis and lipolysis. The expression of lipogenic and lipolytic genes was then analyzed in adipose tissues. The results showed that the mRNA levels of acetyl-CoA carboxylase 2 (*Acc2*) and triglyceride synthetic gene *Dgat1* were increased, while the expression of sterol regulatory element binding transcription factor 1 (*Srebf1*), *Acc1*, *Fasn*, and *Scd1* were not changed by zinc supplementation in the epididymal adipose tissue compared with the control (Figure 5A). The expression of *Srebf1*, *Acc1*, *Acc2*, *Fasn*, *Scd1*, and *Dgat1* was not changed by zinc supplementation in either perirenal or subcutaneous adipose tissue compared with the control mice (Figure 5B,C). Interestingly, the protein level of FASN was much lower in the epididymal adipose tissue of the zinc-supplemented mice than that of control mice (Figure 5D,E). These data demonstrate that short-term zinc supplementation might impair fatty acid synthesis by decreasing the protein expression of FASN in adipose tissue.

### 3.5. Short-Term Zinc Supplementation Promoted the Expressions of Lipolytic Genes and Proteins in the Epididymal Adipose Tissue

**Figure 6:**
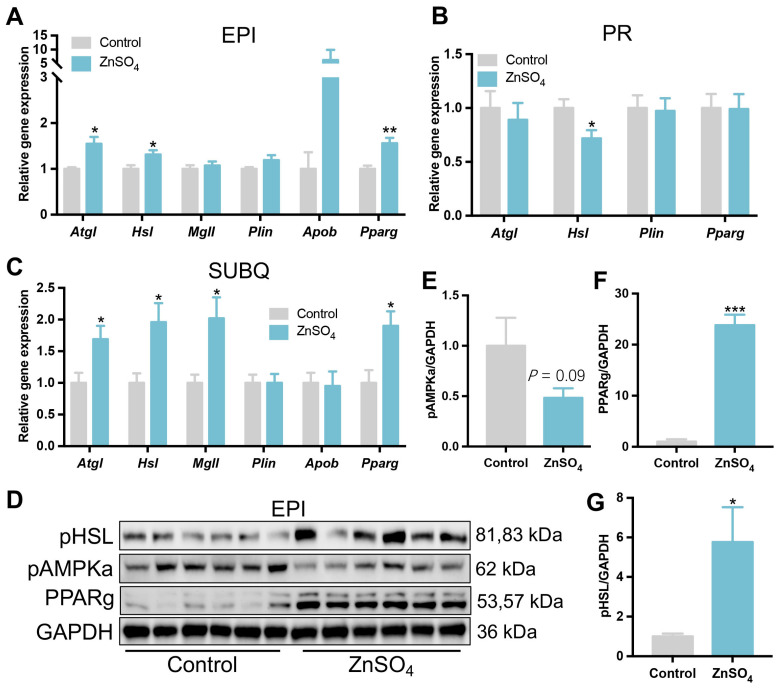
Expression of genes and proteins related to lipid metabolism in adipose tissues. (**A**–**C**) The expression levels of lipid metabolism-related genes in the epididymal (**A**), perirenal (**B**), and subcutaneous (**C**) adipose tissues. (**D**–**G**) Phosphorylation levels of HSL and AMPKa, and protein levels of PPARg in the epididymal adipose tissue (N = 6 for each group). Data are expressed as mean ± SE. ZnSO_4_, 30 ppm zinc supplemented group. * *p* < 0.05, ** *p* < 0.01, *** *p* < 0.001.

Next, the expressions of fat catabolism-related genes were analyzed in the visceral adipose tissue. The results suggested that the mRNA levels of the main enzymes related to lipid catabolism, including adipose triglyceride lipase (*Atgl*), hormone-sensitive lipase (*Hsl*), and *Pparg* were significantly higher in the epididymal adipose tissue of zinc-supplemented mice compared with the control mice; although no change was observed in the expression of monoglyceride lipase (*Mgll*), lipid droplet-associated protein (*Plin*), and apolipoprotein b (*Apob*) between the two groups (Figure 6A). Furthermore, as compared with the control mice, the zinc-supplemented mice had higher expression levels of *Hsl*, *Atgl*, *Mgll*, and *Pparg* in the subcutaneous adipose tissue, while the expression level of *Hsl* in the perirenal adipose tissue was decreased by short-term zinc supplementation, compared with the control mice (Figure 6B,C). Furthermore, the protein level of PPARg and phosphorylation level of HSL in the epididymal adipose tissue of zinc-supplemented mice were increased compared with those in the control mice, while the phosphorylation level of AMPKa tended to be decreased (Figure 6D–G). These data suggest that short-term zinc supplementation could promote lipolysis in epididymal and subcutaneous adipose tissues.

### 3.6. Short-Term Zinc Supplementation Increased the Expressions of Inflammatory Genes in Adipose Tissue

**Figure 7:**
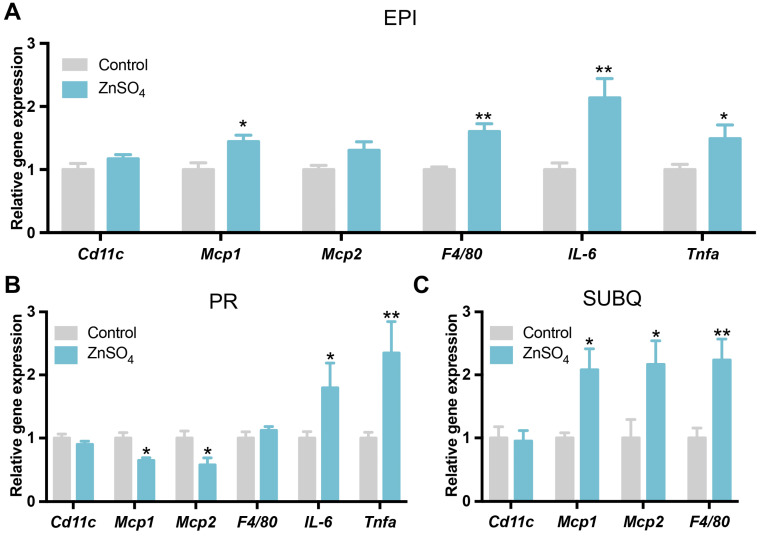
Expression of inflammatory genes in adipose tissues. (**A**–**C**) The expression level of inflammatory genes in the epididymal (**A**), perirenal (**B**), and subcutaneous (**C**) adipose tissues (N = 6 for each group). Data are expressed as mean ± SE. ZnSO_4_, 30 ppm zinc supplemented group. * *p* < 0.05, ** *p* < 0.01.

Previous studies showed that long-term zinc supplementation induced the expression of inflammatory genes in adipose tissue [20]. Here, the expressions of marker genes for inflammatory cytokines and macrophages were analyzed in the adipose tissue. The results showed that short-term zinc supplementation increased the expression of *Tnfa* and *Il6* in both epididymal and perirenal adipose tissues compared with the control group (Figure 7A,B). The mRNA levels of *Mcp1* and *F4/80* were significantly increased in both epididymal and subcutaneous adipose tissues of zinc-supplemented mice as compared to the control mice (Figure 7A,C). However, the expression of *Cd11c* was not changed by zinc supplementation in either of the adipose tissues (Figure 7A–C). These data suggest that short-term zinc supplementation might induce inflammation in adipose tissue.

## 4. Discussion

Micronutrient deficiency is harmful to health, such as impairing growth, increasing the risk of acute infectious disease, causing birth defects, and even death [1,2]. On the other hand, appropriate supplementation of microelement benefits human health [22]. The current study reported that short-term dietary zinc supplementation could regulate lipid metabolism in animals. Firstly, short-term zinc-supplemented mice exhibited lower adipose tissue weight and adipocyte size than the control mice. In addition, short-term zinc supplementation significantly increased blood glucose level and serum insulin concentration, while decreasing the serum NEFA content. Furthermore, the expression of lipolytic genes *Atgl* and *Hsl* were higher in the epididymal and subcutaneous adipose tissue of zinc-supplemented mice than those of the control mice. Meanwhile, the phosphorylation level of HSL and protein of PPARg were increased in the epididymal adipose tissue of zinc-supplemented mice compared with the control mice.

Previous studies found that chronic-zinc supplementation (30 ppm zinc in drinking water for 21 weeks) did not change body weight or food intake, but increased fat deposition in mice [20]. The current study showed that short-term zinc supplementation (30 ppm or 90 ppm zinc in drinking water for one week) did not change the food intake, water consumption, or body weight, but decreased fat deposition. Thus, the decreased adipose tissue weight and adipocyte size seemed not to depend on the feed intake in the current study. In another study with male C57BL/6J mice, zinc supplementation (60 ppm in diet) for 8 weeks did not change the body weight gain or fat weight index under either a normal-chow diet or a high-fat diet [23]. Furthermore, when prepubertal male Swiss mice (5-week-old) were fed with zinc-supplemented diet (25, 50, and 100 mg/kg) for 60 days, 50 and 100 mg/kg zinc-supplemented mice had higher body weight than the control mice [24]. The study by Rech et al. indicated that zinc supplementation (270 mg/kg zinc in the diet for 8 weeks) in Zucker diabetic fatty (ZDF) rats did not alter body weight, epididymal, or visceral fat weight [10]. The different animal models and different treatment durations between different studies might be the reason for the inconsistent results on body weight and fat weight under zinc supplementation. A recent study by Jiang et al. showed that thermogenic adipocytes could secret zinc ion to promote thermogenesis to reduce fat deposition [25]. Another study showed that a high-zinc diet-induced hepatic lipolysis [26]. Studies on humans showed that obese individuals had lower serum zinc concentrations than normal controls [27,28,29]. Thus, in clinic intermittent zinc supplementation might be applied to treat obese men and to reduce fat deposition. However, this should be further explored.

Insulin plays a pivotal role in maintaining glycemia. An increase in blood glucose levels prompts pancreatic islet cells to secrete more insulin, subsequently activating the PI3K/AKT signaling pathway and enhancing the uptake of glucose by adipose tissue and muscle, ultimately resulting in a reduction in the blood glucose level. However, when insulin sensitivity in peripheral tissues is compromised, the hypoglycemic effect of insulin is diminished, giving rise to insulin resistance and hyperinsulinemia [30]. Herein, we showed that zinc-supplemented mice exhibited higher blood glucose levels and serum insulin concentration compared to control mice. Further investigations revealed that short-term dietary zinc intake reduced the phosphorylation level of AKT and inhibited the expression of *Glut4* in the epididymal adipose tissue. GLUT4 plays a key role in maintaining whole-body glucose homeostasis in the adipose tissues and muscle [31,32]. The diminished GLUT4 delivery to the cell surface in the adipose tissue and muscle is the cause of the impairment of insulin-stimulated glucose transport [33,34]. Thus, the increase of blood glucose and insulin levels might be due to the impaired AKT signaling and the expression of *Glut4* in the epididymal adipose tissue of zinc-supplemented mice. Moreover, previous studies revealed that chronic high dose zinc supplementation diminished systemic insulin sensitivity and impaired AKT signaling in the perirenal adipose tissue [20]. Although variations existed in the duration of zinc treatment, these two studies indicated that both long-term and short-term zinc supplementation could impair AKT signaling in adipose tissue, through zinc-enhanced insulin sensitivity in vitro [20]. The increased expression of inflammatory genes in adipose tissue might be the reason for the impairment of insulin sensitivity. It is interesting to figure out the effect of intermittent zinc supplementation on insulin sensitivity and inflammatory gene expression in adipose tissue, which will be explored in future studies.

Fat deposition in the adipose tissue is regulated by lipolysis and lipogenesis [19]. In this study, short-term dietary zinc supplementation reduced adipose tissue weight. It was reported that FASN is the key enzyme for lipogenesis [35]. Here, the protein level of FASN was down-regulated in the epididymal adipose tissue of the zinc-supplemented mice, while the gene expression of *Fasn* was not changed. Previous studies have revealed that chronic high-dose zinc supplementation significantly decreased the protein expression of FASN in the perirenal adipose tissue [20]. Thus, both short-term and long-term zinc supplementation might suppress lipogenesis by reducing the protein expression of FASN in the adipose tissue. However, the exact mechanism for high-dose zinc suppression of FASN protein expression needs further study to be illustrated.

In the case of nutrient scarcity or increased energy requirements, animals undergo lipolysis, which breakdown TAG into NEFAs and glycerol in adipose tissue. ATGL and HSL are the major enzyme for lipolysis in the adipose tissue [36]. In the current study, the mRNA levels of *Atgl* and *Hsl* were significantly increased in the epididymal and subcutaneous adipose tissues of mice receiving short-term dietary zinc supplementation compared to the control mice. These genes are responsible for the central step of TAG lipolysis in the adipose tissue [37]. In this study, the phosphorylation level of HSL was upregulated in the epididymal adipose tissue of zinc-supplemented mice. Consequently, the decreased adipose deposition is probably dependent on lipolysis.

The current study also showed that short-term dietary zinc supplementation decreased the serum NEFA level. NEFA can be used for oxidation to meet the energy requirement of cells [38]. TAG is degraded into NEFA through lipolysis, and a proportion of circulating NEFA originates from visceral adipose tissue. CD36 plays an important role in both fatty acid uptake and the release in adipocytes [39]. Here, the mRNA levels of *Cd36* were decreased in the epididymal, perirenal, and subcutaneous adipose tissues of zinc-supplemented mice. At the same time, the expression of lipolytic gene *Atgl* and *Hsl* were increased by zinc supplementation in epididymal and subcutaneous adipose tissues, and the phosphorylation level of HSL in epididymal adipose tissue was increased, which means an increase in lipolysis in the adipose tissue by zinc supplementation. However, the plasma concentration of NEFA was decreased by zinc supplementation. Thus, short-term zinc supplementation might impair NEFA release by reducing the expression of *Cd36* in adipocytes, which could induce the accumulation of NEFA in adipocytes.

TNFa and IL6 are the main inflammatory factors in the adipose tissue [40]. In the current study, the expression of *Il6* and *Tnfa* was elevated in the visceral adipose tissue of zinc-supplemented mice, suggesting that short-term zinc supplementation may induce inflammation in adipose tissue, which might be a reason for the attenuation of AKT signaling in the epididymal adipose tissue of zinc-supplemented mice. Moreover, we also observed that short-term zinc supplementation stimulated the expression of *F4/80* and *Mcp1* in the epididymal adipose tissue. It has been reported that MCP1 is involved in the recruitment of macrophages in adipose tissue [41]. Macrophage infiltration into adipose tissue also contributes to adipose inflammation and insulin resistance [42]. Thus, the increased inflammation in zinc-supplemented adipose tissue might be due to the accumulation of NEFA in adipocytes and the infiltration of macrophages.

## 5. Conclusions

In summary, this study revealed that short-term zinc supplementation might reduce fat deposition by enhancing lipolysis in mice. Future studies could focus on the effect of intermittent zinc supplementation on fat reduction in both animal models and humans.

## Figures and Tables

**Table 1:** Primers for real-time quantitative PCR.

Genes	Forward	Reverse
*Actb*	GGCTGTATTCCCCTCCATCG	CCAGTTGGTAACAATGCCATGT
*Acc1*	CGGACCTTTGAAGATTTTGTCAGG	GCTTTATTCTGCTGGGTGAACTCTC
*Acc2*	GGAAGCAGGCACACATCAAGA	CGGGAGGAGTTCTGGAAGGA
*Apob*	TTGGCAAACTGCATAGCATCC	TCAAATTGGGACTCTCCTTTAGC
*Atgl*	CTGTGTGGAACCAAAGGACCTG	GCTACCCGTCTGCTCTTTCATC
*Cd11c*	CTGGATAGCCTTTCTTCTGCTG	GCACACTGTGTCCGAACTCA
*Cd36*	ATGGGCTGTGATCGGAACTG	GTCTTCCCAATAAGCATGTCTCC
*Dgat1*	TCCGTCCAGGGTGGTAGTG	TGAACAAAGAATCTTGCAGACGA
*Fasn*	GGCTCTATGGATTACCCAAGC	CCAGTGTTCGTTCCTCGGA
*F4/80*	TGACTCACCTTGTGGTCCTAA	CTTCCCAGAATCCAGTCTTTCC
*Glut4*	ACCGGATTCCATCCCACAAG	TCCCAACCATTGAGAAATGATGC
*Hsl*	TGAAGCCAAAGATGAAGTGAGAC	CTTGACTATGGGTGACGTGTAGAG
*Il6*	TAGTCCTTCCTACCCCAATTTCC	TTGGTCCTTAGCCACTCCTTC
*Leptin*	GAGACCCCTGTGTCGGTTC	CTGCGTGTGTGAAATGTCATTG
*Mcp1*	TTAAAAACCTGGATCGGAACCAA	GCATTAGCTTCAGATTTACGGGT
*Mcp2*	CCCTTCGGGTGCTGAAAAG	CCACTTCTGTGTGGGGTCTAC
*Mgll*	CGGACTTCCAAGTTTTTGTCAGA	GCAGCCACTAGGATGGAGATG
*Pgc1a*	TATGGAGTGACATAGAGTGTGCT	CCACTTCAATCCACCCAGAAAG
*Plin*	CGTGGAGAGTAAGGATGTCAATG	GGCTTCTTTGGTGCTGTTGTAG
*Pparg*	GGAAGACCACTCGCATTCCTT	TCGCACTTTGGTATTCTTGGAG
*Scd1*	CCTACGACAAGAACATTCAATCCC	CAGGAACTCAGAAGCCCAAAGC
*Srebf1*	AACTGCCCATCCACCGACTC	ATTGATAGAAGACCGGTAGCGC
*Tnfa*	GACCCTCACACTCAGATCATCTTCT	CCACTTGGTGGTTTGCTACGA

**Table 2:** Effect of short-term zinc supplementation on body weight and tissue weight.

	Control	30 ppm ZnSO_4_	90 ppm ZnSO_4_	*p* Value
Body weight (g)	28.682 ± 0.282	29.100 ± 0.343	28.767 ± 0.388	0.6823
Tissue weight (g)				
Liver weight	1.282 ± 0.065	1.425 ± 0.025	1.353 ± 0.023	0.1364
Epididymal fat	0.428 ± 0.018	0.345 ± 0.033	0.348 ± 0.024	0.0704
Perirenal fat	0.158 ± 0.010 ^a^	0.105 ± 0.015 ^b^	0.114 ± 0.012 ^b^	0.0248
Subcutaneous fat	0.304 ± 0.018 ^a^	0.206 ± 0.024 ^b^	0.232 ± 0.013 ^b^	0.0099
Sum of white fat depots	0.890 ± 0.045 ^a^	0.656 ± 0.064 ^b^	0.695 ± 0.046 ^b^	0.0184
Brown fat	0.062 ± 0.002	0.061 ± 0.001	0.052 ± 0.002	0.1930
Tissue weight index (%)				
Liver weight	4.469 ± 0.222	4.898 ± 0.075	4.707 ± 0.087	0.2080
Epididymal fat	1.493 ± 0.057 ^a^	1.182 ± 0.108 ^b^	1.214 ± 0.088 ^b^	0.0496
Perirenal fat	0.549 ± 0.031 ^a^	0.360 ± 0.051 ^b^	0.398 ± 0.044 ^b^	0.0200
Subcutaneous fat	1.057 ± 0.060 ^a^	0.710 ± 0.084 ^b^	0.808 ± 0.046 ^b^	0.0076
Sum of white fat depots	3.099 ± 0.143 ^a^	2.253 ± 0.217 ^b^	2.421 ± 0.171 ^b^	0.0135
Brown fat	0.214 ± 0.006	0.210 ± 0.019	0.182 ± 0.007	0.1590

Note: Tissue weight index = (tissue weight/body weight) × 100%. Data are presented as means ± SE. (N = 6 per group). ^a,b^ Values within a row with different superscript letters were significantly different (*p* < 0.05).

## Data Availability

Data are available on request from the corresponding author.
